# Maize inoculation with aflatoxigenic and biocontrol fungi - toxin transfer from feed into milk and yoghurt

**DOI:** 10.1007/s12550-026-00634-4

**Published:** 2026-02-23

**Authors:** Julika Lamp, Karin Knappstein, Christine Schwake-Anduschus, Stefan Nöbel, Janine Saltzmann, Sven Dänicke, Markus Schmidt-Heydt, Ronald Maul

**Affiliations:** 1 Max Rubner- Institut, Federal Research Institute of Nutrition and Food, Institute of Safety and Quality of Milk and Fish Products, Kiel, Germany; 2 Max Rubner- Institut, Federal Research Institute of Nutrition and Food, Institute of Safety and Quality of Cereals, Detmold, Germany; 3 Friedrich- Loeffler- Institut, Federal Research Institute for Animal Health, Institute of Animal Nutrition (ITE), Braunschweig, Germany; 4 Max Rubner- Institut, Federal Research Institute of Nutrition and Food, Institute of Safety and Quality of Fruit and Vegetables, Karlsruhe, Germany

**Keywords:** Biocontrol strains, Aspergillus, Trichoderma, AFB_1_, AFM_1_, Degradation

## Abstract

The consumption of milk can contribute to the consumers` exposure to aflatoxin as aflatoxin M_1_ (AFM_1_), a hydroxylated metabolite of aflatoxin B_1_ (AFB_1_), is transferred into the milk of AFB_1_ exposed dairy cows. As mitigation measures against AFB_1_ presence in cattle feed, fungal biocontrol strains are applied on plant materials to reduce toxigenic *A. flavus* growth. The present study investigates the impact of applying two biocontrol strains (*Trichoderma afroharzianum* and non-toxigenic *Aspergillus flavus*) compared with a toxigenic *A. flavus* strain on the mycotoxin transfer from maize into milk and milk products in a “farm to fork” approach. Firstly, in a feeding study three groups of high yielding dairy cows are exposed to different maize flours infested with biocontrol or AFB_1_-foming fungal strains for 14 days to monitor a potential toxin transfer from feed to milk. In the milk of cows exposed to AFB_1_, AFM_1_ was detected with a mean value of (95.6 ng/kg ± 13.7 ng/kg) while the mean transfer rates (3.8% ± 0.9%) were slightly higher than published in previous studies. Neither AFM_1_ nor cyclopiazonic acid could be detected in milk from the cows exposed to the two biocontrol strains. The data show, that the AFM_1_ limit of 50 ng/kg in milk can be exceeded when cattle feed is contaminated with AFB_1_ within the legal limit. Consequently, the use of cattle feed within the current European legal limits does not necessarily guarantee for obtaining milk compliant with European maximum levels. In the second part, AFM_1_ containing raw milk from the experimental cows was processed further into yoghurt to investigate the fate of the toxin under acidic fermentation conditions. No AFM_1_ degradation was found during authentic yoghurt making, albeit in a model experiment the hydration of AFM_1_ in presence of citric acid was shown. Further studies are necessary to investigate the exact conditions suitable for reducing the AFM_1_ content of contaminated milk during the processing of dairy products

**Supplementary Information:**

The online version contains supplementary material available at 10.1007/s12550-026-00634-4.

## Introduction

The awareness that fungal spoilage may be harmful to human and animal health exists for thousands of years along with the recognition that measures must be taken to prevent such health risks. Regarding the feed and food chain, these measures can be taken at different target points like feed production and storing, feeding management and food processing. Strains of the *Aspergillus* (*A.*) genus are known to produce mycotoxins, such as the well-known carcinogenic aflatoxin B_1_ (AFB_1_), which are of particular toxicological relevance. Aflatoxins are produced on a wide variety of food and feed commodities in the field and during storage, predominantly in hot and humid regions. Maize is one of the most important staple foods worldwide and an important habitat for aflatoxin forming *Aspergilli* at the same time. Reducing fungal infection or toxin formation are key approaches to prevent loss of entire batches of feed or food. Besides classical synthetic fungicides there is a need to find effective and safe non-fungicide natural eco-friendly alternatives to control fungal decay (Droby 2006). Several approaches to utilise antagonistic microorganisms for biocontrol of agricultural products have been applied, including the use of *Saccharomyces cerevisiae* or lactic acid bacteria in food preservation (Muñoz, 2010) as well as using *Bacillus cereus* to control the growth of *Aspergillus* species on peanut kernels (Kumar, 2014). The utilisation of non-aflatoxigenic *A. flavus* strains appears to be suitable for a reduction of aflatoxins, whilst the formation of the mycotoxin cyclopiazonic acid (CPA), which is also known to be transferred from feed to milk (Dorner, 1994), is not minimised. Alternative approaches are also tested in order to identify fungal strains, that are (so far) not known to produce emerging mycotoxins or their precursors. One such approach could be the use of biocontrol fungi, that do not merely compete passively for available space and nutrients in the habitat, but actively combat the undesirable mycotoxin-producing or plant-pathogenic fungal species and do not produce any known mycotoxins themselves. Suitable isolates meeting these requirements can be found in the genus *Trichoderma*, e.g. *Trichoderma afroharzianum* (Braun, 2018).

Besides the direct exposure to the carcinogenic aflatoxins AFB_1_ and aflatoxin G_1_ (AFG_1_), also the main bovine AFB_1_ metabolite in milk (AFM_1_) is responsible for human exposure, if milk of dairy animals is consumed which have been fed with AFB_1_ contaminated feed (EFSA (EFSA 2004)). Mean transfer rates of around 2.0% to 3.0% in high yielding cows under typical European husbandry conditions were determined (Fink-Gremmels 2008; Walte, 2022). Although the metabolite´s toxicity is clearly lower compared to its parent compound AFB_1_ (Wogan and Paglialunga 1974; Prandini, 2009), this toxin requires particular attention due to the potential exposure of infants to AFM_1_ containing milk. Legal limits for AFB_1_ in feed and food as well as for AFM_1_ in milk have been established in many countries (Egmond and Jonker 2003). In the EU, the legal limit of AFB_1_ in dairy cow feedstuffs has been set at 5 µg/kg dry matter (EC- 2002). Complying these limits, it is assumed, that the MRL for AFM_1_ in milk will not be exceeded.

Milk is often processed further with a main focus on fermented milk products in African countries. For lactic acid bacteria a binding of aflatoxins has been discussed earlier (Khadivi, 2020). As yoghurt production goes along with the application of lactic acid bacteria and acidification of the medium, these two factors might lead to a reduction of the aflatoxin burden of the final food. For AFB_1_, it has been shown that a hydration occurs when the toxin is treated with organic acids in aqueous solutions which may also lead to a reduction of the toxic potential (Rushing and Selim 2016). The use of organic acids on AFB_1_ is designed to break the 8,9-double bond, the chemical region responsible for its carcinogenicity. The study by Rushing and Selim investigated the transformation of an AFB_1_ standard (300 ng/mL) in 1 M citric acid solution at room temperature showing that > 90% of the initial AFB1 disappeared after 72 h (Fig. 3). Applying heat of HCl as alternative acid accelerated the hydration of AFB_1_ and also ascorbic and formic acid induced the transformation to AFB_2a_.

AFB_2a_ did not show any reversion to the parent compound after being transferred to a neutral solution, thus a potential reduction of toxicity presumably is permanent. As the AFM_1_ molecule contains the same structural domain a similar reactivity is possible, however, this has not been confirmed so far. Also, for AFB_1_ the exact reaction conditions or potential matrix influences on the reaction have not been clarified systematically. Thus, for both effects, i.e. binding or hydration, it remains unclear whether authentic conditions of yoghurt production are suitable for a real reduction of AFM_1_ burden in contaminated milk.

Controlling the human dietary exposure to aflatoxins with special focus on the carcinogenic AFB_1_, AFG_1_ and AFM_1_ is one promising approach for the reduction of the dietary induced cancer events worldwide. Therefore, the present study aims to investigate safety and efficacy of the AFB_1_/AFM_1_ reduction at different target points along the feed and food chain using a two-part “from farm to fork” approach. In the first part, maize infested with two biocontrol fungal strains (*Trichoderma afroharzianum* and non -toxigenic *Aspergillus flavus*) and a toxigenic *A. flavus* strain was used for a feeding study with 16 dairy cows to investigate the strain-dependant mycotoxin transfer with a special focus to potentially masked aflatoxins. So far, no distinct modifications for AFB_1_ have been described. However, plant induced metabolism i.e. by glycosylation as it is well known for deoxynivalenol (Berthiller, 2011), cannot be ruled for AFB_1_, potentially leading to deliberation of additional free AFB_1_ in the course of the digestion process in the cow. In the second part, the AFM_1_ containing milk from the experimental cows was processed to yoghurt monitoring the toxins` stability under acidic conditions in order to test the hypothesis of toxin binding and degradation processes due to lactic acid bacteria fermentation.

## Materials and methods

### Animal study

Conventional grain maize was obtained from a local supplier. The aflatoxigenic *Aspergillus flavus* strain MRI_19_ (Schamann, 2022) and the biocontrol strain *Trichoderma afroharzianum* MRI_349_ (Braun, 2018) were both obtained from the culture collection of the Max Rubner-Institut. The non-aflatoxigenic *A. flavus* strain AF_36_ (ATCC 96045, NRRL 18543) was obtained from the American Type Culture Collection. The *A. flavus* strain MRI_19_ represented a model strain for a common aflatoxin-producing *A. flavus* strain from the environment. For the inoculation of the feeding maize under controlled conditions, three different fungal strains were used: two biocontrol strains (*T. afroharzianum* and a non-aflatoxigenic *A. flavus*) and an *A. flavus* strain known to produce aflatoxins.

Prior to inoculation, the maize was humidified with sterile water for 3 days at 6 °C and subsequently autoclaved to avoid interference by naturally occurring microorganisms on the kernels. The maize (ten portions of approx. 1 kg per inoculation strain) was plated out in open plastic bags in an incubator in a layer of approx. 5–10 cm and inoculated with 15 mL of a spore solution (spore density of approx. 10^6^ spores/mL) per portion of one of the three different fungal strains. The maize was incubated for seven days at 25 °C; shaken three times during the period to assure aeration and homogenous growth and additionally humidified with sterile water once. After the incubation period, the maize samples were autoclaved and dried for 5 days at 65 °C to avoid uncontrolled further fungal growth. Afterwards, the dried maize grains were ground to 0.5 mm using a mill (Mühlomat 100; Treffler Maschinenbau GmbH & Co. KG, Pöttmes-Echsheim, Germany) and mixed with a standard concrete mixer to achieve homogeneity. Subsequently, the homogeneity of the entire batches was tested by analysis of twelve random samplings of 2.5 g. The results showed a satisfactory homogeneity with content of 2.285 ± 231 µg/kg measured for AFB_1_ only in the batch inoculated with toxigenic *A. flavus*. A subsample of approx. 1 kg of each batch was used for preparation of the boli and analysed in triplicate again (Table 1).

**Table 1:** Aspergillus toxins quantified by HPLC-MS/MS in the inoculated maize and total mixed ratio

	AFB_1_	AFB_2_	CPA	AFB_1_	AFB_2_	CPA
Matrix/Group	µg/kg ± SD			µg uptake/day/animal		
total mixed ration **TMR**	< LOD	< LOD	< LOD	< LOD	< LOD	< LOD
Biocontrol *Trichoderma afroharzianum* **BCT**	< LOD	< LOD	< LOD	< LOD	< LOD	< LOD
Biocontrol *A. flavus* **BCA**	< LOD	< LOD	1836.3 ± 45.6	< LOD	< LOD	73.5
*A. flavus* (toxigenic) **ATox**	2283.6 ± 133.5	285.8 ± 10.8	1005.9 ± 41.2	91.3	11.4	40.2

### Animals 

The experiment was conducted at the agricultural research station of the Max Rubner-Institut in Schädtbek. Overall, 16 lactating, healthy, non-gravid dairy cows, breed German Holstein black and white were allocated to four groups based on the respective performance parameters as shown in *Table SI* 1: one control group (group 1: CON) and three experimental groups (group 2: biocontrol non-aflatoxigenic *A. flavus* (BCA), group 3: biocontrol *T. afroharzianum* (BCT) and group 4: AFB_1_-forming *A. flavus* (ATox)).

During the trial, experimental and control cows were kept together in a separate section of an outdoor climate free stall barn with deep straw bedding cubicles. All animals had free access to a total mixed ration (TMR) containing grass and maize silage, concentrate feed and minerals with an energy content of approx. 6.97 MJ/kg DM. The total feed intake of the group was determined by weighing the daily feed submission and the feed remains on the following day. All cows were milked in a tandem milking parlour (GEA Farm Technologies, Bönen, Germany) twice per day at 7 a.m. and at 5 p.m., corresponding to milking intervals of 10 and 14 h. The animal health status and performance parameters (behaviour, feed intake, rumination, milk yield) were checked by a veterinarian every day throughout the whole trial. The data are given in table SI 5 and 6.

The animal experiment was approved by the Ministry of Energy, Agriculture, the Environment, Nature, and Digitalization of Schleswig-Holstein, Germany (reference number V244-47221/2020; 70 − 8/20). The research adheres to the ARRIVE guidelines and the 3Rs principles. Animals were monitored daily for signs of pain and distress, and any signs of distress were addressed according to a pre-defined protocol.

#### Supplementation

Maize flour was administered orally using capsules (Science Services GmbH, Munich, Germany) prepared for the three experimental groups (one group per fungal treatment). Capsules were filled with 20.0 ± 0.1 g of the maize flour with a target AFB_1_ content of approx. 50 µg per capsule in the ATox group. Animals in the BCA and BCT groups received capsuled containing a comparable amount of the respective maize flour. The control group did not receive a placebo as the daily administered amount of maize flour was considered too small to have any measurable effect on the animal’s health and performance. The supplementation was carried out for 14 days using a rumen bolus applicator (Senior, Vuxxx GmbH, Papenburg, Germany). The cows received one capsule twice a day, given after the morning milking and after the evening milking, respectively. Thus, the daily AFB_1_- dose per cow in the ATox- group corresponded to an intake of 20 kg dry matter (DM) contaminated with the legal limit of 5 µg AFB_1_/kg DM. The precise amounts of mycotoxin exposure are given in Table 1. The supplementation period was followed by a depuration period of 21 days.

#### Milk sampling

The milk of each individual cow was sampled separately at both milking times after thorough mixing of composite milk. The sampling for mycotoxin analysis was carried out once before the beginning of the supplementation (day − 7), during the supplementation period (daily from day 1 to day 14) and the depuration period (days 15, 16, 17, 18, 21 and 35). Each sample was split into two 100 mL aliquots for the mycotoxin analysis. One was frozen at −20 °C and retained, and the second fresh sample was stored for up to 60 h at 4 °C until further analysis. On two separate days (days 11 and 12 of the trial), approx. 40 L of milk was pooled from the morning and evening milk of each of the four groups and used for yoghurt preparation. In total, sixteen milk samples were obtained from the four feeding groups.

### Milk processing

#### Raw milk

Raw milk was collected as described above and daily transferred to the research dairy Kiel (Max Rubner-Institut, Kiel, Germany). All milk was pasteurized (72 °C; 18 s) through a plate heat exchanger (300 L/h; APV Rosista GmbH, Unna, Germany) and kept in cold storage (6 °C) for a maximum of 24 h. For the production of yoghurt, the fat and protein contents were not further adjusted. For the aflatoxin exposed group (ATox), the fat content was 4.6 ± 0.2 g/100 g and protein content was 3.8 ± 0.0 g/100 g (calculated as arithmetical mean with standard deviation); for the other three experimental groups the fat and protein contents were in a very similar range (CON: fat content 4.6 ± 0.2 g/100 g, protein content 3.6 ± 0.1 g/100 g; BCA: fat content 4.3 ± 0.2 g/100 g, protein content 3.5 ± 0.2 g/100 g; BCT: fat content 4.7 ± 0.2 g/100 g, protein content 3.7 ± 0.2 g/100 g).

## Manufacture of yoghurt

Fermented products were made from pasteurized milk with additional heat treatment as shown in a process flow diagram in Appendix Figure SI 1. Using a UHT pilot plant (120 L/h, APV Pasilac A/S, Silkeborg, Denmark) a quantity of 10 kg milk was preheated to 60 °C, homogenized (250/50 bar), high heated to 92 °C for 5 min, immediately cooled to 15 °C, and stored (6 °C) for another 1–2 days. Milk was split into two 4 kg-batches, fermented with either of two commercially available starter cultures supplied by Danisco Deutschland GmbH (Niebüll, Germany):

Yo-Mix 601 resulted in a yoghurt of traditional style, with strong flavour and high viscosity and contained *Streptococcus salivarius* ssp. *thermophilus* and *Lactobacillus delbrueckii* ssp. *bulgaricus.*

Yo-Mix 215 resulted in a yoghurt of mild style, with mild flavour and medium viscosity and consisted of *Streptococcus salivarius* ssp. *thermophilus*,* Lactobacillus delbrueckii* ssp. *lactis*, *Lactobacillus acidophilus* and *Bifidobacterium animalis* ssp. l*actis.*

Two 9 L-vessels, each containing 4 kg of milk, were heated and held at 42.0 °C in parallel using the same water bath (type AEJ 4–10; Altmann Analytik GmbH & Co. KG, Munich, Germany). For fermentation, 20 g starter culture/100 L was added and gently mixed. Temperature and pH were recorded (DAQ Factory Express v19.1; AzeoTech Inc., Ashland, USA) with pre-calibrated pH sensors (SE555X/2-NMSN; Knick Elektronische Messgeräte GmbH & Co. KG, Berlin, Germany). The fermentation (approx. 6 h) was stopped at pH 4.50 by gently stirring the yoghurt, filling and sealing into 150 g-containers, and transfer to a cold-storage room < 10 °C in 30 min. All yoghurt samples were prepared in duplicate, i.e. starting from independent milk samples of different days of the same animals (32 samples in total), and stored at 10 °C for six days before freezing and long-term storage at −20 °C until analysis.

### AFM_2a_ Preparation 

In order to test the hypothesis, that AFM_1_ undergoes the same modification as AFB_1_ and to obtain a reference substance corresponding to AFB_2a_ as acidification induced hydration product of AFB_1_, AFM_1_ was treated as described by Rushing et al. (Rushing and Selim 2016) for the hydration of AFB_1_ with slight modification. One mL AFM_1_ solution (1 µg/mL) in acetonitrile was directly mixed with 1 mL citric acid solution (3% v/v). After 4.5 h of incubation at 60 °C the resulting solution was mixed with approx. 200 mg MgSO_4_ to obtain a phase separation. The organic layer was evaporated to dryness and dissolved in a water methanol mixture (80/20, v/v), diluted by a factor of 1000 and analysed by HPLC-MS/MS.

## Analysis

Depending on the matrix and mycotoxins of interest, different extraction procedures and HPLC-MS/MS analyses were carried out.

### Analysis of feed samples

#### Multi-mycotoxins screening of inoculated maize samples

Triplicate portions of 10 g of each supplement were shaken for one hour with 50 mL of a mixture of acetonitrile/water/acetic acid (79/20/1, v/v/v). A volume of 5 mL of the centrifuged extracts was filtered (0.2 μm regenerated cellulose, Wicom, Germany) prior to analysis. Aliquots of 10 µL were analysed in duplicate to screen the following mycotoxins: aflatoxin B_1_, B_2_, G_1_, G_2_, deacetoxyscirpenol, 15-monoacetoxyscirpenol, ochratoxin A, zearalenone, alpha-zearalenol, beta-zearalenol, zearalanone, alpha-zearalanol, beta-zearalanol (Merck/Sigma-Aldrich, Germany); alternariol, alternariol-methyl-ester, altenuene, altertoxin, tentoxin I, tenuazonic acid (Cfm Oskar Tropitzsch GmbH, Germany); CPA, acetyl-deoxynivalenol, deoxynivalenol, deoxynivalenol-3-glucosid, de-epoxy-deoxynivalenol, ergotalkaloids, fumonisin B_1_, fumonisin B_2_, T2-toxin, HT2-toxin (Romer Labs, Germany) and citrinin (AdipoGen Life Science, Germany).

#### Aflatoxin B_1_, B_2_, G_1_ and G_2_ quantification in maize samples by Stable Isotope Dilution Analysis HPLC-MS/MS 

The samples were weighed to the nearest 0.1 g in triplicate (10 g) for analysis. Then 40 mL of a mixture of acetonitrile/water (84/16, v/v) was added to the maize sample. This mixture was shaken on a horizontal shaker for 30 min (GFL 3020, Gesellschaft für Labortechnik GmbH, Germany) and filtered using cellulose folded filter paper (Whatman, GE Healthcare, United Kingdom). The extract was purified using a cartridge (MycoSep 226 AflaZon, Romer Labs, Germany). The cleaned extract was diluted 1:1000 with a mixture of 5 mmol/L ammonia acetate in water (MilliQ Advantage, Merck, Germany) and 10 µL of internal standard solution (^13^C_17_ -Aflatoxin B_1_ standard 1 ng/mL, Romer Labs, Germany) was added prior to analysis by HPLC-MS/MS.

#### HPLC-MS/MS method for maize analysis

Separation, screening and quantification were performed by high performance liquid chromatography (LC-20 Prominence system, Shimadzu, Germany) coupled to mass spectrometry (4000QTrap, Sciex, Germany). The chromatography was carried out on a Luna Phenyl Hexyl column (5 μm, 150 mm x 2.00 mm, Phenomenex, Germany) at 30 °C. A volume of 10 µL was injected and the flow rate was set to 0.4 mL/min with a gradient programme: starting with 95% A (5 mmol ammonia acetate in water/methanol/acetic acid 89/10/1, v/v/v) and 5% B (5 mmol ammonia acetate/methanol/acetic acid 2/97/1, v/v/v) for 1 min, a linear gradient within 4 min to 95% B, hold to 9 min and returned to 5% B at 9.5 min, hold until 15 min (Table SI 2).

#### Total mixed ration (TMR) analysis 

TMR samples of approx. 500 g were taken during the exposure periods every 2–3 days and stored at −20 °C until freeze drying for further sample preparation. For monitoring of a potential background contamination of the feed, freeze dried homogenized samples of the TMR were analysed for presence of additional aflatoxins and CPA. The TMR was weighed to the nearest 0.1 g in duplicate (2.5 g) and 10 mL water/formic acid (98/2, v/v) were added, mixed and left to settle for 5 min. 10 mL of a mixture of acetonitrile/formic acid (98/2, v/v) and 10 mL n-heptane were added. After vigorous shaking for 15 min and centrifugation for 5 min at 2.300 x g at room temperature, the n-heptane phase was removed and discarded. 10 mL of the remaining mixture were transferred into a new vessel and 0.5 g NaCl and 2.0 g MgSO_4_ were added for phase separation. After shaking and centrifugation as described before, 4.5 mL of the organic phase were transferred into a new tube. 100 µL dimethylsulfoxid as keeper agent were added and the organic solvent was evaporated at 45 °C. Subsequently, 400 µL mobile phase A (water/formic acid (98/2, v/v)) was added and vortexed. The sample was filtered by regenerated cellulose, 13 mm, 0.45 μm syringe filter.

The limits of detection (LOD) for the feed analysis were derived from spiking regular samples apparently free of AFB_1_ and CPA with toxins levels in the range of the expected limit of quantification (LOQ). The samples were treated as described before and analysed by HPLC-MS/MS. The LOD was defined as the threefold signal-to-noise ratio of the quantifier transition in a threefold analysis of a spiked blank matrix sample including extraction and clean-up. The LOD was calculated as 0.15 µg/kg for AFB_1_ and 27 µg/kg for CPA in the TMR (Figure SI 2, A and B).

For the quantification of CPA in the inoculated maize samples, the same protocol for the TMR was used, and additionally, prior to the addition of NaCl and MgSO_4_, 100 µL of internal standard (^13^C_20_- CPA, 100 ng/mL, Romer Labs, Germany) were added.

### Milk and yoghurt analysis 

For milk analysis, 5 mL of the samples were measured in duplicate. For yoghurt analysis, 2.5 g were mixed with 2.5 mL water. 100 µL of both internal standards ^13^C_17_-aflatoxin M_1_ (c = 1.5 ng/mL) and 100 µL of ^13^C_20_- CPA (c = 50 ng/mL, both of Romer Labs, Germany), were added. 5 mL of a mixture of acetonitrile/formic acid (99/1, v/v) and 5 mL n-heptane were added and after shaking and centrifugation fat was removed with the heptane phase. Phase separation, concentration and sample filtration were performed as described for TMR samples with the exception that only 4.0 mL of organic sample phase were evaporated. The residue was resolved in 100 µL mobile phase B and vortexed. After 5 min ultrasonication 400 µL mobile phase A was added and vortexed. The samples were filtered with regenerated cellulose, 13 mm, 0.45 μm syringe filter prior to analysis.

#### HPLC-MS/MS method for TMR, milk and yoghurt analysis 

Separation and quantification were performed by ultrahigh performance liquid chromatography (1290 system, Agilent, USA) coupled to mass spectrometry (QTrap6500+, Sciex, Germany). The measurements were carried out on an Poroshell 120 C18 column (2.1 × 50 mm, 1.9 μm, Agilent, USA) at 35 °C. A volume of 5 µL was injected and the flow rate was set to 0.4 mL/min with a gradient programme: starting at 20% B (5 mmol ammonium formate in methanol/water/formic acid (98.9/1/0.1, v/v/v) for 0.5 min, a linear gradient within 4.5 min to 95% B, hold to 7 min, direct increase to 20% B hold until 10 min. Eluent A was 5 mmol ammonium formate in water/formic acid (99.9/0.1, v/v).

The LOD was derived from threefold spiking milk sample apparently free of AFM_1_ at a toxin level of 5 ng/L milk, i.e. in the range of the expected LOQ. The LOD was defined as the threefold signal-to-noise ratio of the quantifier transition and calculated to be 1.4 ng AFM_1_/L. Accordingly, for CPA an LOD of 46 ng/L was calculated in milk and yoghurt.

### Statistical analysis and calculations 

**Fig. 1:**
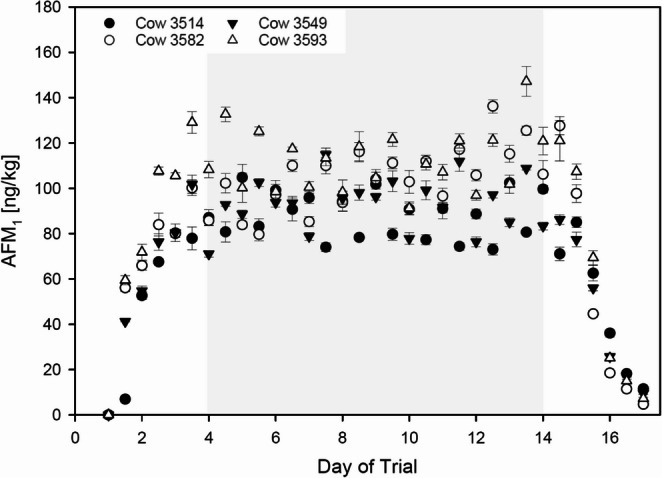
AFM_1_content in the milk samples of the ATox exposed group measured by HPLC-MS/MS plotted for each milking time separately. The last bolus was given after milking in the evening of day 14. Error bars in the graph describe the variance (SD) of three independent measurements

**Fig. 2:**
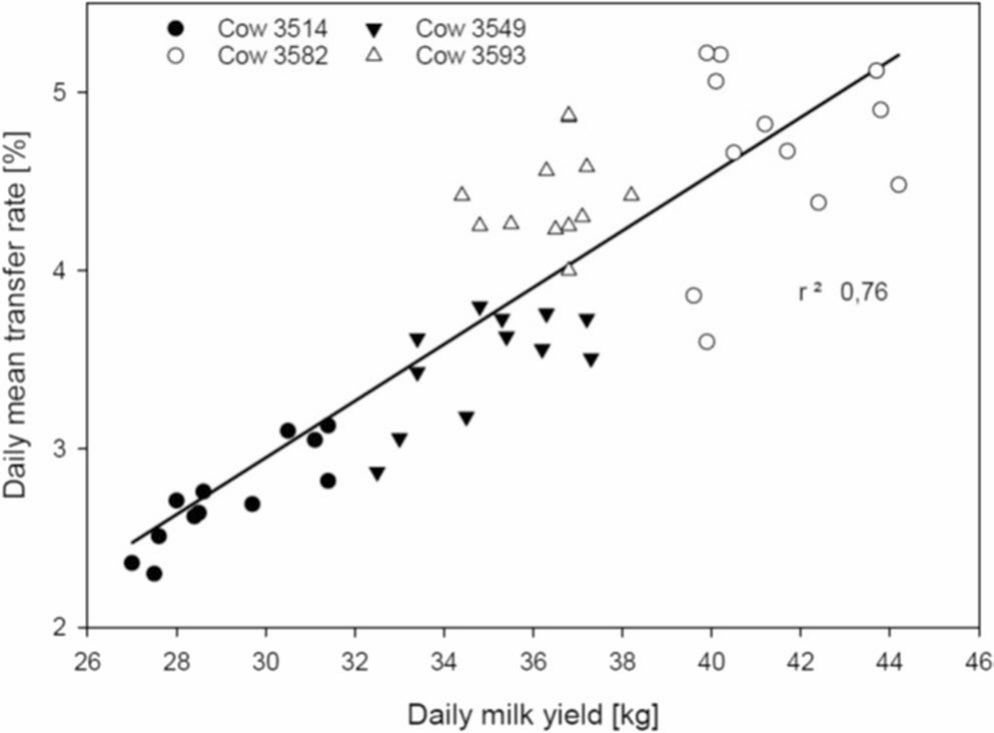
Correlation of the daily mean aflatoxin transfer rates in the steady-state phase (day 4 to 14, as indicated in Figure1) of the four AFB_1_ exposed cows to the daily milk yield. A linear regression can be derived (y = 0.1591 x – 1.8205; with x = milk yield and y = transfer rate) with r² = 0.76

Figures 1 and 2 were evaluated using Statistica (TIBCO Software Inc. (2020). Data Science Workbench, version 14). The transfer rate from feed to milk refers to the proportion of a contaminant present in animal feed that is excreted in the milk produced by exposed animals and was calculated according to the following equation:$\:TR\left[\%\right]=\frac{DailyExcretionViaMilk\left[\frac{\mu\:g}{d}\right]}{DailyIntakeViaFeed\left[\frac{\mu\:g}{d}\right]}\times\:100\%$ 

## Results and discussion 

### Contaminated feed

The treatment of feed maize with fungi resulted in an intense AFB_1_ and AFB_2_ production in the case of *A. flavus* (ATox). No aflatoxins were detectable with the biocontrol strains BCA and BCT under the same conditions (Table 1). Toxigenic strain ATox as well as the non-aflatoxigenic biocontrol strain BCA resulted in the formation of CPA. CPA was absent in samples treated with BCT. The screening analysis of the inoculated maize batches did not show any additional signals for the investigated mycotoxins except trace amounts of FB_1_ and ZEN that can be attributed to a background contamination of the initial feed maize (Table SI 3).

The maximum acceptable level of AFB_l_ in compound feed for dairy cattle is 5 µg/kg, based on a dry matter content of 88% (EC (EC 2011)). Calculated on the basis of a typical daily feed intake of approx. 20 kg dry matter by dairy cows, an amount of 100 µg AFB_1_ per day was derived as the theoretical maximum value that could be contributed by feed within the legal limits. Consequently, two boli containing 20 g ground maize of each inoculum were used per day in the trial, resulting in a final daily uptake of 91.3 µg AFB_1_ (Table 1). As the TMR did not contain any AFB_1_ or CPA above the LOQ, additional mycotoxin intake from the cows´ regular diet was neglectable. Basic animal health- and performance parameters were monitored daily throughout the entire feeding trial. However, none of these parameters were altered by treatment. Therefore, it can be concluded that neither the short-term daily AFB_1_ exposure of approx. 100 µg/animal nor the exposure to maize kernels inoculated with two different biocontrol fungal strains lead to obvious adverse effects on the observed health parameters. However, effects on the internal organs or gene expression levels need to be assessed separately.

### Milk analysis 

No mycotoxins were found in milk samples taken before the start of the trial in any of the groups. Aflatoxins were detected only in the ATox group when milk samples from the trial period were analysed (Figure SI 3). CPA was not detected in the milk of any of the feeding groups, although the ATox and the BCA group had daily intakes of 40.2 µg and 73.5 µg of CPA, respectively (Table 1).

As CPA was not detected above the LOD, a maximum transfer rate was calculated based on the total amount of intake and a theoretical concentration in milk. Considering the LOD of 0.046 µg/L for CPA in the applied method used for milk analysis, which was not exceeded, and considering the daily supplementation of 73.5 µg for the BCA group, a transfer of the total amount of toxin would have resulted in an approximate content of 2 µg/L in the milk (based on a medium milk yield of approx. 35 L/day). As no signal for CPA was detected in milk, it can be assumed that the transfer rate of unmetabolized CPA does not exceed 2.3%. In this case, previous reports of the presence of more than 1 µg/L CPA in milk may either be attributed to a very high contamination level of the TMR fed to the cows or there exist other origins than the feed-to-milk transfer of the CPA contamination (Oliveira, Sebastião et al. (Oliveira, 2008)). In addition, Ye et al. (Ye, 2024) published data on the in vitro metabolites of CPA obtained in microsomal incubations, revealing that the metabolism of CPA includes dehydrogenation, hydroxylation, methylation, and glucuronidation, with hydroxylation being the primary metabolic fate. Due to the lack of standards for the metabolites, it was not possible to confirm or exclude the formation of these compounds in vivo in the present study. However, two expected mass transitions were included in the mass spectrometric analysis that did not give detectable signals in any of the milk samples. The theoretical mass transitions for the hydroxylated CPA were calculated by adding 16 amu to the precursor mass and by measuring the main CPA fragments once with and without the mass shift of 16 amu compared to CPA using the same ESI source parameters as for CPA.

After the third day of the trial, the measured AFM_1_ levels in milk seemed to reach a steady state, resulting in AFM_1_ evels in milk between 71.1 ng/kg and 147.2 ng/kg (Fig. 1). Thus, starting with day four, the data were included for further evaluation in the steady-state. Individual data are given in table SI 4. Based on these data and the daily milk yield, individual daily transfer rates for AFB_1_ to AFM_1_ in milk were calculated (Table SI 4). The daily intake of 91 µg AFB_1_ per animal resulted in mean individual transfer rates that varied from 2.72 to 4.67% between the four individuals, with an arithmetic mean of about 3.8%.

As illustrated in Fig. 2, our data showed a tendency towards higher transfer rates with higher daily milk yield for the AFB_1_ transfer and transformation into AFM_1_. Although the four individuals examined in the present study represent a very limited number, the data are consistent with the findings of Costamagna et al. (Costamagna, 2019), where transfer rates were more than twice as high in high yielding cows than in low yielding ones. Similar results were reported by Jonathan et al. (Jonathan, 2024) with only slightly higher transfer in higher yielding cows. In a study based on multi-year data collection, Costamagna et al. (Costamagna, 2021) have shown that different components of the dairy cattle diet can contain considerable levels of aflatoxins with silages, pastures, commercial feeds, and cotton seed being the ingredients most correlated with the aflatoxin levels in milk. Therefore, the total diet has to be considered when AFB_1_ intake is discussed in the context of the compliance with the legal limit in milk.

Overall, the data from our study show that, in a worst-case scenario considering a feed regime with AFB_1_ levels well below the 5 µg/kg limit in various components of the total cows´ diet, the concentration of 50 ng/kg milk can be exceeded. This result is also in line with a previous study by Xu et al. (Xu, 2021) who reported mean AFM_1_ levels in raw milk between 70 and 130 ng/kg, while in the TMR, also collected from the farms, AFB_1_ levels ranged from 3.9 to 4.6 µg/kg. Thus, the present study confirms in a controlled trial the results previously obtained in a field trial (Xu, Xiao et al. 2021).

### Analysis of milk products produced from naturally contaminated milk 

#### AFM_1_ to AFM_2a_ transformation and analysis 

**Fig. 3:**
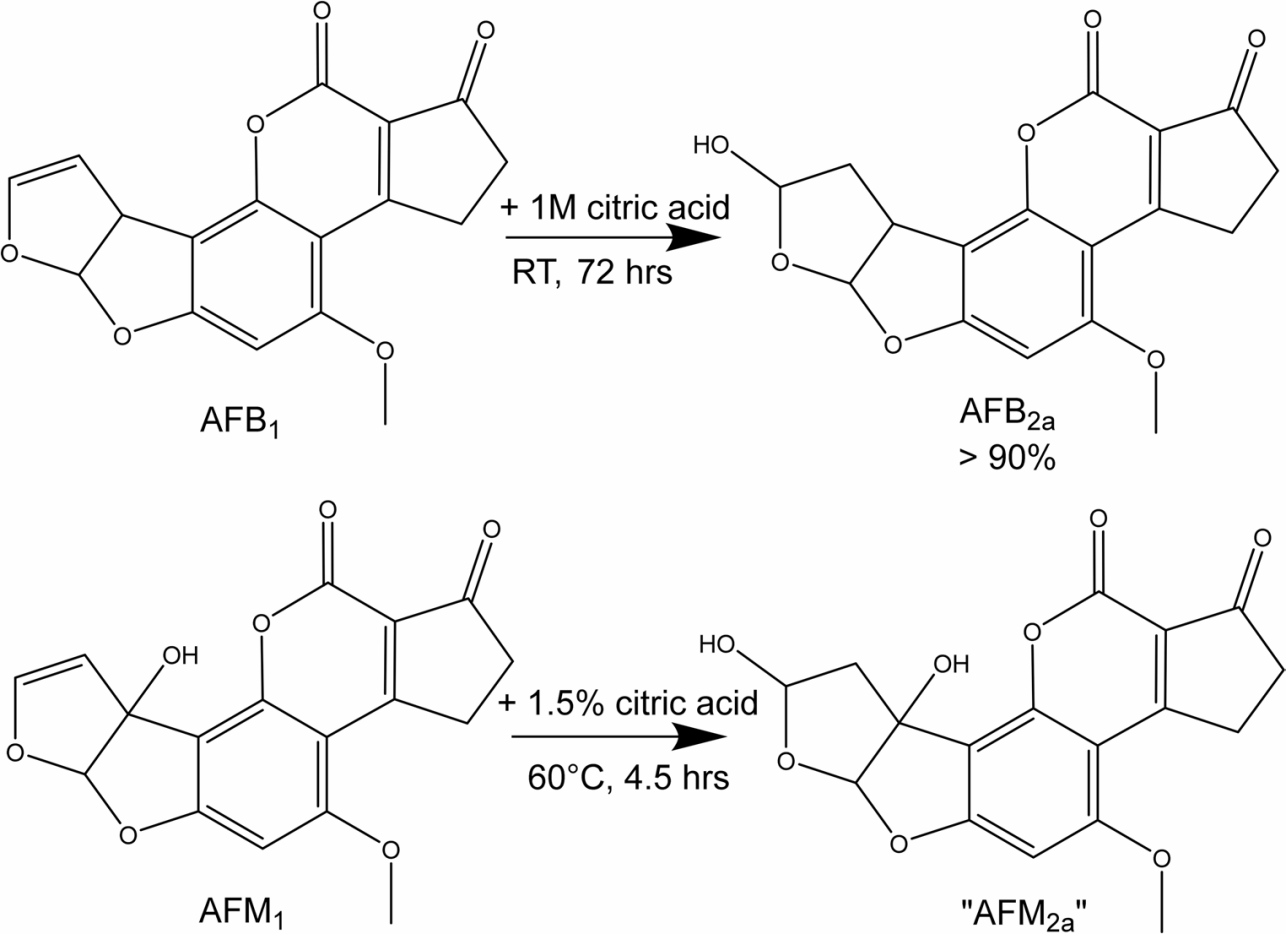
Hydration occurring during organic acid treatment of AFB_1_ according to Rushing and Selim (2016) (top) and the proposed analogous formation of AFM_2a_ as an acid-induced degradation product of AFM_1_ (bottom)

To test the stability of AFM_1_ under moderately acidic conditions, the toxin was first treated with organic acid under aqueous conditions in a model experiment. Treatment of AFM_1_ in H_2_O/acetonitrile (50/50, v/v) at 60 °C in 1.5% citric acid results in a decrease in AFM_1_ signal intensity of approx. 50% compared to the control sample, which was only heat treated but without citric acid. To facilitate the sample clean-up and analysis, the citric acid concentration was lower than in the reference experiment performed by Rushing et al. (Rushing and Selim 2016) where AFB_1_ was almost quantitatively converted to AFB_2a_ using 1 M acid (equivalent to approx. 20% w/v), which is almost ten times higher than in our study. Monitoring of postulated mass transitions matching to the fragments observed for AFB_2a_ plus the additional hydroxy function in AFM_1_ in the HPLC-MS/MS analysis shows the appearance of an additional peak at a retention time which is 1.2 min shorter (Figure SI 4). As these mass transitions based on a molecular weight of AFM_1_ with an addition of 18 amu are absent in the control experiment and the double bond in the moiety of AFM_1_ is the only site susceptible to an easy acid-induced addition of a water molecule, we highly suggest the formation of the AFM_2a_ structure as shown in Fig. 3.

Thus, treatment of AFM_1_ with citric acid provides an indication for the hydration of the substance, similar to the transformation of AFB_1_ to AFB_2a_, even under relatively mild conditions using only 1.5% of citric acid. However, without further analysis (NMR, hrMS) there is no clear evidence for the AFB_2a_-like structure as hypothesised in Fig. 3. Nevertheless, the simple model experiment shows that AFM_1_ is also susceptible to acid-induced degradation.

#### AFM_1_ stability during yoghurt Preparation 

As a transformation of AFM_1_ could be demonstrated in the aqueous model experiment, it was investigated whether similar processes take place during acidification in yoghurt production. Yoghurt analysis revealed an almost quantitative stability of AFM_1_ during production, as the recovery of AFM_1_ in both yoghurt types in all replicates is almost complete compared to the raw milk obtained from the transfer study. The AFM_1_ content of approx. 100 ng/L from naturally contaminated raw milk was also detected in yoghurt in the range between 89 ng/L and 114 ng/L (Table 2). Similar processing of milk artificially contaminated to contain 880 ± 90 ng/L of AFM_1_ after pasteurisation resulted in the detection of 620 ng/L in yoghurt samples (Daou, 2025), corresponding to a transfer factor of 69%. Concentrating this yoghurt by filtration increased AFM_1_ accordingly, indicating that AFM_1_ had become attached to the protein network during fermentation.

**Table 2:** Aflatoxin M_1_ (AFM_1_) content in samples taken during processing of Raw milk into yoghurt (mean and standard deviation of three independent analyses)

	1 st pool milk of cows 3514 and 3582		2nd pool milk of cows 3514 and 3582		1 st pool milk of cows 3549 and 3593		2nd pool milk of cows 3549 and 3593	
sample type	mean [ng/L]	SD [ng/L]	mean [ng/L]	SD [ng/L]	mean [ng/L]	SD [ng/L]	mean [ng/L]	SD [ng/L]
raw milk	107.6	3.6	100.3	2.5	92.2	2.6	99.3	1.5
high-heated milk	113.2	3.9	101.8	1.9	94.9	1.9	98.5	2.8
Yo-Mix 601^*)^	109.4	5.3	95.5	2.8	93.2	2.5	96.8	2.2
Yo-Mix 215^*)^	113.9	4.9	101.3	5.4	88.9	6.9	91.0	1.1

*) Yoghurt produced with either YoMix 601 (traditional style) or YoMix 215 (mild style) starter culture

If binding of the toxin occurs, as discussed in previous model studies (Khadivi, 2020), according to the present study such an effect only leads to a very small reduction and is not reproducible in all experiments. Furthermore, no hydration is observed under the conditions of yoghurt production process used. Although, a hydration was preliminarily demonstrated and corresponding mass transitions could be derived for the HPLC-MS/MS analysis, no such mass spectrometric signals were observed in authentic yoghurt samples.

### Overall discussion and conclusion 

A positive correlation trend between milk yield and AFM_1_ transfer rate was observed. Together with climate change, which is associated with improved growth conditions for aflatoxin forming fungi on crops such as maize, increasing milk yield in dairy cows may increase the frequency and levels of AFM_1_ in the future. However, the increasing occurrence of AFB_1_ is at least as worrying, as recent studies have shown that the contribution of AFM_1_ from milk to the global incidence of liver cancer is small and the parent compound AFB_1_ is likely to be of greater concern (Saha Turna, 2022). Comparing the transfer of AFB_1_ to AFM_1_ from feed to milk with previous data, the transfer rates obtained in this study, ranging from 2.72% to 4.67%, are slightly higher than in the study by Walte et al. (2.3% to 2.5%) (Walte, 2022). Since the milk yields in both studies were in a similar range, the approximately 50% increase in transfer rates (from 2.4% to 3.8%) cannot be explained by the observed effect of increasing transfer rates with higher milk yields. However, it is possible that the naturally contaminated material used in this study contained additional masked forms of AFB_1_, that could not be detected by the targeted HPLC-MS/MS analysis applied. Therefore, the actual AFB_1_ levels may have been higher than in the study by Walte et al. (Walte, 2022) who worked with spiked material. In addition, the higher exposure level of 91 µg/day in the present study compared to 50 µg/day or the fact that two boli were administered daily could also have an impact on the transfer rate.

Due to the toxic effects of aflatoxins, especially AFB_1_, but also AFM_1_, where contamination scenarios leading to an exceedance of the legal limit in milk have been shown to be realistic, measures to control and reduce toxin formation are desirable. According to the available data, no adverse effects on performance and health parameters of dairy cattle were observed for maize treated with the two biocontrol fungi tested. Furthermore, no transfer of the accompanying mycotoxin CPA was detected in the milk. If proven effective under field conditions, the application of non-aflatoxigenic *A. flavus* and *T. afroharzianum* could be a promising approach to safely reduce AFB_1_ exposure of dairy cattle and the transfer of AFM_1_ in milk.

Under the conditions chosen for this transfer study, it can be assumed that the applied biocontrol strains do not have a negative effect on the safety of milk from exposed dairy cows. As no transfer to milk was observed, the emerging mycotoxin CPA appears to be of minor importance as a contaminant in feed after biocontrol application. Nevertheless, since *T. afroharzianum* is unable to produce the mycotoxin CPA, unlike non-aflatoxigenic *A. flavus*, its application as a biocontrol is more advisable from this point of view.

While the exposure of dairy cattle to AFB_1_ by feed – within the current European legal limits – had no detectable adverse effects on the animals, the AFM_1_ limit of 50 ng/kg in milk was exceeded when the entire feed ratio was equally contaminated with AFB_1_, even within its legal limit. Consequently, the use of cattle feed within the current European legal limits does not necessarily guarantee for obtaining milk a compliant with European maximum levels. A trend towards a positive correlation between milk yield and transfer rate could be deduced, but this needs to be confirmed in a larger study. As under comparable experimental settings transfer rates of AFB_1_ from feed to AFM_1_ in milk have been found to be approx. 50% higher for naturally contaminated maize (3.8% ± 0.9%) compared to feed containing AFB_1_ spiked as pure reference compound (2.4% ± 0.7%) (Walte, 2022), either additional AFB_1_-like masked compounds not covered or extracted by conventional methods are present or the resorption of AFB_1_ is enhanced for maize matrix exposed to *Aspergillus* fungi. Either way, care should be taken when transfer rate data from different experimental settings are compared. Furthermore, AFM_1_ was shown to be susceptible to acid degradation into AFM_2a_, but the conditions of yoghurt production were not sufficient. Further studies are needed to investigate whether other food processing techniques are suitable for toxicity reduction by mild to moderate organic acid treatment or if the conditions are too harsh to be relevant.

## Supplementary Information

Supplementary Material 1

## Data Availability

No datasets were generated or analysed during the current study.

## Abbreviations

ATox: AFB_1_-forming Aspergillus flavus group
BCA: biocontrol non-aflatoxigenic Aspergillus flavus group
BCT: biocontrol Trichoderma afroharzianum group
CON: control group
CPA: Cyclopiazonic acid
HPLC-MS/MS: High-Performance Liquid Chromatography-Tandem Mass Spectrometry
