# Correction to: Maize inoculation with aflatoxigenic and biocontrol fungi - toxin transfer from feed into milk and yoghurt

**DOI:** 10.1007/s12550-026-00648-y

**Published:** 2026-04-20

**Authors:** Julika Lamp, Karin Knappstein, Christine Schwake-Anduschus, Stefan Nöbel, Janine Saltzmann, Sven Dänicke, Markus Schmidt-Heydt, Ronald Maul

**Affiliations:** 1 Max Rubner- Institut, Federal Research Institute of Nutrition and Food, Institute of Safety and Quality of Milk and Fish Products, Kiel, Germany; 2 Max Rubner- Institut, Federal Research Institute of Nutrition and Food, Institute of Safety and Quality of Cereals, Detmold, Germany; 3 Friedrich- Loeffler- Institut, Federal Research Institute for Animal Health, Institute of Animal Nutrition (ITE), Braunschweig, Germany; 4 Max Rubner- Institut, Federal Research Institute of Nutrition and Food, Institute of Safety and Quality of Fruit and Vegetables, Karlsruhe, Germany

**Correction to: Mycotoxin Research (2026) 42:26**

10.1007/s12550-026-00634-4

In this article, the annotated corrections were not implemented, and the provided supplementary file was not utilized.

The original article has been corrected.

## Supplementary Information

Below is the link to the electronic supplementary material.

Supplementary Material 1 (DOC 6.90 mb)

