# Supplementary material for: Correction to: Maize inoculation with aflatoxigenic and biocontrol fungi - toxin transfer from feed into milk and yoghurt
Source: Mycotoxin Res. 2026 Apr 20;42(2):38. doi: 10.1007/s12550-026-00648-y (PMC13095901; doi:10.1007/s12550-026-00648-y)
Supplement: Supplementary file 1 — Supplementary Material 1 (DOC 6.90 mb) [file 12550_2026_648_MOESM1_ESM.doc]

**Supplementary material**

**Table SI 1:** Performance parameters of the experimental cows.

| Animal number | Fungal strain | Number of lactation | Days in milk ^1^ | Milk yield  (kg/d) ^2^ | Body weight  (kg) ^3^ |
| --- | --- | --- | --- | --- | --- |
| **CON** | x |  |  |  |  |
| 3450 |  | 4 | 135 | 40.6 | 720 |
| 3585 |  | 2 | 157 | 38.4 | 700 |
| 3589 |  | 2 | 63 | 37.3 | 685 |
| 3599 |  | 2 | 134 | 41.1 | 700 |
| Mean ± SD |  | 2.5 ± 1.0 | 122 ± 41 | 39.4 ± 1.6 | 701 ± 14 |
| **BCA** | *Aspergillus flavus*  non-aflatoxigenic |  |  |  |  |
| 3492 |  | 4 | 75 | 47.7 | 715 |
| 3529 |  | 3 | 208 | 32.6 | 740 |
| 3590 |  | 2 | 149 | 34.2 | 675 |
| 3602 |  | 2 | 130 | 33.2 | 640 |
| Mean ± SD |  | 2.8 ± 1.0 | 141 ± 55 | 36.9 ± 6.2 | 693 ± 44 |
| **BCT** | *Trichoderma afroharzianum* |  |  |  |  |
| 3506 |  | 4 | 112 | 30.2 | 750 |
| 3586 |  | 2 | 164 | 34.9 | 690 |
| 3587 |  | 2 | 141 | 40.1 | 695 |
| 3588 |  | 2 | 67 | 42.4 | 645 |
| Mean ± SD |  | 2.5 ± 1.0 | 121 ± 42 | 36.9 ± 4.7 | 695 ± 43 |
| **ATox** | *Aspergillus flavus*  toxigenic |  |  |  |  |
| 3514 |  | 3 | 144 | 31.6 | 685 |
| 3549 |  | 3 | 181 | 36.8 | 815 |
| 3582 |  | 2 | 104 | 42.5 | 725 |
| 3593 |  | 2 | 124 | 37.2 | 720 |
| Mean ± SD |  | 2.5 ± 0.6 | 138 ± 33 | 37.0 ± 3.9 | 736 ± 55 |

Days relative to start of supplementation: ^1^ day 1, ^2^ mean value days -1 to -6, ^3^ days -1 to -14

**Table SI 2: Parameter for different analytes in the multi reaction mode (MRM) for mass spectrometry**

| **Analyte** | **Q1 mass [Da]** | **Q3 mass [Da]** | **Dwell time [msec]** | **DP**  **[V]** | **CE**  **[V]** | **CXP**  **[V]** |
| --- | --- | --- | --- | --- | --- | --- |
| AFB_1_ | 312.98 | 241.10 | 80 | 91 | 51 | 18 |
|  |  | 284.80 | 100 | 91 | 33 | 22 |
| AFB_2_ | 314.96 | 259.10 | 100 | 76 | 41 | 20 |
|  |  | 286.90 | 100 | 76 | 37 | 24 |
| AFG_1_ | 328.99 | 242.90 | 100 | 71 | 39 | 18 |
|  |  | 310.90 | 100 | 71 | 31 | 24 |
| AFG_2_ | 331.00 | 313.00 | 10 | 121 | 33 | 42 |
|  |  | 245.10 | 10 | 121 | 41 | 10 |
| AFM_1_ | 328.90 | 273.00 | 100 | 116 | 33 | 16 |
|  |  | 229.00 | 100 | 116 | 53 | 26 |
| ^13^C-AFB_1_ | 330.07 | 255.00 | 100 | 86 | 51 | 14 |
|  |  | 138.30 | 100 | 86 | 95 | 22 |
| ^13^C-AFM_1_ | 345.90 | 288.00 | 80 | 116 | 33 | 16 |
|  |  | 242.00 | 80 | 116 | 53 | 26 |

| **Analyte** | **Q1 mass [Da]** | **Q3 mass [Da]** | **Dwell time [msec]** | **DP**  **[V]** | **CE**  **[V]** | **CXP**  **[V]** | **EP**  **[V]** |
| --- | --- | --- | --- | --- | --- | --- | --- |
| CPA | 335.2 | 140 | 100 | -130 | -40 | -7 | -10 |
|  |  | 154 | 100 | -130 | -46 | -11 | -10 |
| ^13^C_20_-CPA | 355.2 | 146 | 100 | -130 | -40 | -7 | -10 |
|  |  | 165 | 100 | -130 | -46 | -11 | -10 |

**Table SI 3**: The inoculated maize samples were screened by HPLC-MS/MS for undesired mycotoxins: HT2-toxin [75], T2-toxin [75], AFB_1_[7.5], AFB_2_[7.5], AFG_1_[7.5], AFG_2_[7.5], acetyl-deoxynivalenol [75], de-epoxy-deoxynivalenol [75], 15-monoacetoxyscirpenol [3], deacetoxyscirpenol [37.5], ergotalkaloids (ergo(E)metrin [6], E metrinin [6], E sin [6], E sinin [6], E cornin [6], E corninin [6], E tamin [6], E taminin [6], E cryptin [6], E cryptinin [6], E cristin [6], E cristinin [6]), ochratoxin A [6], citrinin [11.25], deoxynivalenol [25], zearalenone [2.5], alpha-zearalenol [2.5], beta-zearalenol [2.5], zearalanone [2.5], alpha-zearalanol [2.5], beta-zearalanol [2.5], deoxynivalenol-3-glucosid [60], fumonisin B_1_ [2.5], fumonisin B_2_ [5], altenuene [5], alternariol-methyl-ester [5], alternariol [2.5], altertoxin I [12.5], tentoxin [12.5], tenuazonic acid [50]; [LOQ is given in brackets in µg/kg]). All maize samples were analysed in triplicate. Mycotoxin concentrations above the LOQ are given in the table.

|  | **LOD; LOQ** | **Group BCA** | **Group ATox** | **Group BCT** |
| --- | --- | --- | --- | --- |
|  |  | *A. flavus*, MRI 351 | *A. flavus*, MRI 019 | *T. afroharzianum*, MRI 349 |
|  | [µg/kg] | [µg/kg] | [µg/kg] | [µg/kg] |
| AFB_1_ | 2.5; 7.5 | <LOD | 550 | <LOD |
| AFB_2_ | 2.5; 7.5 | <LOD | 60 | <LOD |
| CPA | 50; 150 | 1570 | 1100 | <LOD |
| Zearalenone | 0.8; 2.5 | 20 | 5 | 10 |
| Fumonisin B_1_ | 0.8; 2.5 | 6 | 8 | 7 |

LOD = 1/3 LOQ

**Table SI 4: AFM_1_ contents in milk samples of group ATox, individual milk yields and transfer rates (TR)**

| **Day** | **Animal number** | **Milking time** | **Afla M1 ng/kg** | **CV in %** | **Milk yield [kg]** | **Daily AFM1 amount excreted via milk [µg]** | **Daily TR [%]** |
| --- | --- | --- | --- | --- | --- | --- | --- |
| 1 | 3514 | 1 | n.d. | n.a. | 18.2 |  |  |
| 1 | 3514 | 2 | 52.7 | 3.3 | 12.6 | 0.8 | n.a. |
| 1 | 3549 | 1 | n.d. | n.a. | 14.8 |  |  |
| 1 | 3549 | 2 | 41.3 | 0.7 | 15.7 | 0.6 | n.a. |
| 1 | 3582 | 1 | n.d. | n.a. | 26.0 |  |  |
| 1 | 3582 | 2 | 56.1 | 2.9 | 17.0 | 1 | n.a. |
| 1 | 3593 | 1 | n.d. | n.a. | 22.4 |  |  |
| 1 | 3593 | 2 | 59.4 | 3.3 | 13.5 | 0.8 | n.a. |
| 2 | 3514 | 1 | 67.6 | 1.8 | 18.6 |  |  |
| 2 | 3514 | 2 | 80.3 | 1 | 12.9 | 2.3 | 2.5 |
| 2 | 3549 | 1 | 54.8 | 3.8 | 21.6 |  |  |
| 2 | 3549 | 2 | 76.3 | 4.6 | 15.5 | 2.4 | 2.6 |
| 2 | 3582 | 1 | 65.9 | 2.9 | 25.4 |  |  |
| 2 | 3582 | 2 | 84.0 | 5.8 | 17.4 | 3.1 | 3.4 |
| 2 | 3593 | 1 | 71.8 | 4.8 | 22.0 |  |  |
| 2 | 3593 | 2 | 107.7 | 1.7 | 14.8 | 3.2 | 3.5 |
| 3 | 3514 | 1 | 78.0 | 6.3 | 18.1 |  |  |
| 3 | 3514 | 2 | 87.1 | 3.9 | 13.3 | 2.6 | 2.8 |
| 3 | 3549 | 1 | 80.4 | 4.8 | 20.5 |  |  |
| 3 | 3549 | 2 | 101.8 | 3.8 | 15.7 | 3.2 | 3.6 |
| 3 | 3582 | 1 | 80.0 | 1.9 | 22.4 |  |  |
| 3 | 3582 | 2 | 100.2 | 3.3 | 17.3 | 3.5 | 3.9 |
| 3 | 3593 | 1 | 105.6 | 1.3 | 22.5 |  |  |
| 3 | 3593 | 2 | 129.1 | 3.6 | 13.8 | 4.2 | 4.6 |
| 4 | 3514 | 1 | 80.8 | 5.3 | 18.0 |  |  |
| 4 | 3514 | 2 | 104.9 | 5.3 | 13.4 | 2.9 | 3.1 |
| 4 | 3549 | 1 | 71.1 | 2.1 | 18.1 |  |  |
| 4 | 3549 | 2 | 92.8 | 0.8 | 14.4 | 2.6 | 2.9 |
| 4 | 3582 | 1 | 85.8 | 2.4 | 25.9 |  |  |
| 4 | 3582 | 2 | 102.2 | 4.1 | 18.3 | 4.1 | 4.5 |
| 4 | 3593 | 1 | 108.3 | 3.2 | 21.9 |  |  |
| 4 | 3593 | 2 | 132.7 | 2.4 | 12.5 | 4 | 4.4 |
| 5 | 3514 | 1 | 83.2 | 4 | 18.8 |  |  |
| 5 | 3514 | 2 | 99.2 | 4.2 | 12.3 | 2.8 | 3.1 |
| 5 | 3549 | 1 | 88.6 | 2 | 20.7 |  |  |
| 5 | 3549 | 2 | 102.5 | 1.6 | 15.6 | 3.4 | 3.8 |
| 5 | 3582 | 1 | 83.9 | 1.8 | 25.8 |  |  |
| 5 | 3582 | 2 | 79.6 | 3.5 | 14.2 | 3.3 | 3.6 |
| 5 | 3593 | 1 | 100.2 | 6.2 | 22.5 |  |  |
| 5 | 3593 | 2 | 125.1 | 1.5 | 13.0 | 3.9 | 4.3 |
| 6 | 3514 | 1 | 90.7 | 5.4 | 18.3 |  |  |
| 6 | 3514 | 2 | 95.9 | 2.6 | 12.2 | 2.8 | 3.1 |
| 6 | 3549 | 1 | 93.8 | 2.1 | 21.7 |  |  |
| 6 | 3549 | 2 | 93.6 | 2.9 | 13.7 | 3.3 | 3.6 |
| 6 | 3582 | 1 | 97.2 | 2.2 | 26.7 |  |  |
| 6 | 3582 | 2 | 110.1 | 2.3 | 17.1 | 4.5 | 4.9 |
| 6 | 3593 | 1 | 98.5 | 3.2 | 23.3 |  |  |
| 6 | 3593 | 2 | 117.5 | 0.6 | 13.8 | 3.9 | 4.3 |
| 7 | 3514 | 1 | 74.0 | 2.6 | 17.4 |  |  |
| 7 | 3514 | 2 | 94.4 | 4.6 | 12.3 | 2.4 | 2.7 |
| 7 | 3549 | 1 | 78.7 | 2.2 | 24.1 |  |  |
| 7 | 3549 | 2 | 114.9 | 2.5 | 13.1 | 3.4 | 3.7 |
| 7 | 3582 | 1 | 85.3 | 2.5 | 26.6 |  |  |
| 7 | 3582 | 2 | 110.0 | 3.3 | 15.8 | 4 | 4.4 |
| 7 | 3593 | 1 | 100.5 | 2.5 | 23.0 |  |  |
| 7 | 3593 | 2 | 113.3 | 2.9 | 13.8 | 3.9 | 4.3 |
| 8 | 3514 | 1 | 78.3 | 0.9 | 16.9 |  |  |
| 8 | 3514 | 2 | 101.8 | 1.9 | 11.7 | 2.5 | 2.8 |
| 8 | 3549 | 1 | 95.6 | 2.2 | 20.7 |  |  |
| 8 | 3549 | 2 | 98.2 | 3.3 | 14.6 | 3.4 | 3.7 |
| 8 | 3582 | 1 | 93.7 | 4.1 | 25.8 |  |  |
| 8 | 3582 | 2 | 116.2 | 3.5 | 16.0 | 4.3 | 4.7 |
| 8 | 3593 | 1 | 98.3 | 5.3 | 22.6 |  |  |
| 8 | 3593 | 2 | 118.3 | 5.4 | 13.9 | 3.9 | 4.2 |
| 9 | 3514 | 1 | 79.7 | 3.2 | 16.7 |  |  |
| 9 | 3514 | 2 | 90.8 | 2.7 | 11.7 | 2.4 | 2.6 |
| 9 | 3549 | 1 | 96.3 | 1.6 | 21.1 |  |  |
| 9 | 3549 | 2 | 103.2 | 4.3 | 12.3 | 3.3 | 3.6 |
| 9 | 3582 | 1 | 104.0 | 3.9 | 25.8 |  |  |
| 9 | 3582 | 2 | 111.3 | 2.2 | 17.9 | 4.7 | 5.1 |
| 9 | 3593 | 1 | 104.8 | 2.3 | 21.1 |  |  |
| 9 | 3593 | 2 | 121.6 | 2.5 | 13.7 | 3.9 | 4.3 |
| 10 | 3514 | 1 | 77.3 | 2.8 | 16.4 |  |  |
| 10 | 3514 | 2 | 91.1 | 4.9 | 11.2 | 2.3 | 2.5 |
| 10 | 3549 | 1 | 77.9 | 3.2 | 23.5 |  |  |
| 10 | 3549 | 2 | 99.2 | 4.1 | 13.8 | 3.2 | 3.5 |
| 10 | 3582 | 1 | 102.9 | 4.7 | 23.9 |  |  |
| 10 | 3582 | 2 | 112.0 | 2.2 | 17.3 | 4.4 | 4.8 |
| 10 | 3593 | 1 | 91.3 | 3 | 21.7 |  |  |
| 10 | 3593 | 2 | 110.7 | 2.3 | 15.1 | 3.7 | 4 |
| 11 | 3514 | 1 | 74.4 | 2.1 | 16.9 |  |  |
| 11 | 3514 | 2 | 88.6 | 2.4 | 10.1 | 2.2 | 2.4 |
| 11 | 3549 | 1 | 91.6 | 2.4 | 21.2 |  |  |
| 11 | 3549 | 2 | 112.0 | 3.9 | 13.6 | 3.5 | 3.8 |
| 11 | 3582 | 1 | 96.6 | 3.5 | 23.9 |  |  |
| 11 | 3582 | 2 | 117.2 | 3.2 | 16.6 | 4.3 | 4.7 |
| 11 | 3593 | 1 | 107.1 | 3.2 | 23.0 |  |  |
| 11 | 3593 | 2 | 121.0 | 2.5 | 14.2 | 4.2 | 4.6 |
| 12 | 3514 | 1 | 73.0 | 3.3 | 17.4 |  |  |
| 12 | 3514 | 2 | 102.4 | 1.3 | 11.1 | 2.4 | 2.6 |
| 12 | 3549 | 1 | 76.4 | 2.7 | 21.3 |  |  |
| 12 | 3549 | 2 | 97.2 | 1.5 | 13.2 | 2.9 | 3.2 |
| 12 | 3582 | 1 | 105.8 | 2.2 | 23.7 |  |  |
| 12 | 3582 | 2 | 136.3 | 2.1 | 16.5 | 4.8 | 5.2 |
| 12 | 3593 | 1 | 96.9 | 2.2 | 24.5 |  |  |
| 12 | 3593 | 2 | 121.3 | 2.2 | 13.7 | 4 | 4.4 |
| 13 | 3514 | 1 | 80.7 | 1.3 | 16.7 |  |  |
| 13 | 3514 | 2 | 99.7 | 1.6 | 11.3 | 2.5 | 2.7 |
| 13 | 3549 | 1 | 85.0 | 2.1 | 21.2 |  |  |
| 13 | 3549 | 2 | 108.8 | 0.7 | 12.2 | 3.1 | 3.4 |
| 13 | 3582 | 1 | 115.1 | 3.1 | 24.0 |  |  |
| 13 | 3582 | 2 | 125.5 | 1.6 | 15.9 | 4.8 | 5.2 |
| 13 | 3593 | 1 | 101.7 | 3.9 | 21.7 |  |  |
| 13 | 3593 | 2 | 147.2 | 4.4 | 15.1 | 4.4 | 4.9 |
| 14 | 3514 | 1 | 71.1 | 4.1 | 17.2 |  |  |
| 14 | 3514 | 2 | 85.0 | 2.6 | 10.3 | 2.1 | 2.3 |
| 14 | 3549 | 1 | 83.3 | 2 | 20.1 |  |  |
| 14 | 3549 | 2 | 86.2 | 2.3 | 12.9 | 2.8 | 3.1 |
| 14 | 3582 | 1 | 106.2 | 5.5 | 23.2 |  |  |
| 14 | 3582 | 2 | 127.7 | 3 | 16.9 | 4.6 | 5.1 |
| 14 | 3593 | 1 | 120.9 | 4.9 | 22.8 |  |  |
| 14 | 3593 | 2 | 121.0 | 7.2 | 14.0 | 4.4 | 4.9 |
| 15 | 3514 | 1 | 62.5 | 5.4 | 17.2 |  |  |
| 15 | 3514 | 2 | 36.1 | 4.3 | 11.6 | 1.5 | 1.6 |
| 15 | 3549 | 1 | 77.4 | 4 | 21.2 |  |  |
| 15 | 3549 | 2 | 56.0 | 2.3 | 12.6 | 2.3 | 2.6 |
| 15 | 3582 | 1 | 97.8 | 3.9 | 22.2 |  |  |
| 15 | 3582 | 2 | 44.7 | 2.5 | 14.5 | 2.8 | 3.1 |
| 15 | 3593 | 1 | 107.4 | 3.2 | 23.7 |  |  |
| 15 | 3593 | 2 | 69.4 | 4.5 | 15.5 | 3.6 | 4 |
| 16 | 3514 | 1 | 18.3 | 4.3 | 17.0 |  |  |
| 16 | 3514 | 2 | 11.6 | 9.7 | 10.5 | 0.4 | 0.5 |
| 16 | 3549 | 1 | 25.7 | 4.1 | 18.8 |  |  |
| 16 | 3549 | 2 | 17.0 | 1.5 | 15.6 | 0.8 | 0.8 |
| 16 | 3582 | 1 | 18.5 | 3.5 | 23.7 |  |  |
| 16 | 3582 | 2 | 11.5 | 3.3 | 15.9 | 0.6 | 0.7 |
| 16 | 3593 | 1 | 25.1 | 3.3 | 23.0 |  |  |
| 16 | 3593 | 2 | 15.0 | 4.7 | 16.1 | 0.8 | 0.9 |
| 17 | 3514 | 1 | 5.7 | 5.7 | 17.1 |  |  |
| 17 | 3514 | 2 | 3.9 | 10.9 | 10.7 | 0.1 | 0.2 |
| 17 | 3549 | 1 | 9.4 | 4.3 | 20.1 |  |  |
| 17 | 3549 | 2 | 7.1 | 2.7 | 13.1 | 0.3 | 0.3 |
| 17 | 3582 | 1 | 4.7 | 4.7 | 23.7 |  |  |
| 17 | 3582 | 2 | n.d. | n.a. | 15.9 | 0.1 | 0.1 |
| 17 | 3593 | 1 | 7.2 | 7 | 22.1 |  |  |
| 17 | 3593 | 2 | 4.7 | 11.3 | 14.1 | 0.2 | 0.3 |
| 18 | 3514 | 1 | n.d. | n.a. | 16.8 |  |  |
| 18 | 3514 | 2 | n.d. | n.a. | 10.5 | n.a. | n.a. |
| 18 | 3549 | 1 | n.d. | / | 22.1 |  |  |
| 18 | 3549 | 2 | n.d. | / | 12.9 | n.a. | n.a. |
| 18 | 3582 | 1 | n.d. | n.a. | 23.7 |  |  |
| 18 | 3582 | 2 | n.d. | n.a. | 15.6 | n.a. |  |
| 18 | 3593 | 1 | n.d. | / | 19.5 |  |  |
| 18 | 3593 | 2 | n.d. | / | 14.7 | n.a. | n.a. |

**n.a.**: not applicable, **n.d**.: not detectable.

**Table SI 5: Median performance parameters of the experimental cows.**

|  | **Milk yield (kg/day)** | | | | | | | | **Feed intake (kg DM/day)** | |
| --- | --- | --- | --- | --- | --- | --- | --- | --- | --- | --- |
| **Group** | **CON** | | **BCA** | | **BCT** | | **Atox** | | **all animals** | |
| **Period** | **Mean** | **SD** | **Mean** | **SD** | **Mean** | **SD** | **Mean** | **SD** | **Mean** | **SD** |
| 1 | 39.5 | 2.0 | 37.1 | 6.5 | 37.1 | 4.9 | 37.0 | 4.1 | 25.8 | 0.8 |
| 2 | 38.4 | 2.2 | 36.3 | 6.0 | 36.2 | 4.5 | 35.6 | 4.6 | 25.4 | 0.8 |
| 3 | 37.0 | 2.4 | 34.7 | 5.8 | 34.4 | 5.2 | 33.1 | 4.6 | 25.5 | 1.0 |

Period 1: preliminary investigation day -7 till -1, period 2: feeding trial day 1 till 14, period 3: depuration period day 15 till 35

SD: standard deviation

**Table SI 6:** Daily scored animal health parameters.

|  | **Daily scoring** | | | |
| --- | --- | --- | --- | --- |
| **Score** | **0** | **1** | **2** | **3** |
| Behaviour | normal | segregation from the group | somnolence | down |
| Feed intake | normal | reduced by 25% | reduced by 50% | no feed intake |
| Rumination | normal | reduced | highly reduced | no rumination |
| Milk yield | normal | reduced by 25% | reduced by 50% | reduced by 100% |

All experimental cows were checked by a veterinarian daily.

The health parameters were scored using a point system from 0= normal,

1= low grade alterations, 2= middle grade alterations, 3= high grade alterations. The system provided for the termination of the exposure if one parameter reached a score of 2 or if the sum of all parameters equalled 3.

**Figure SI 1:** Basic process flow diagram for the manufacture of yoghurt from the raw milk of the feeding trial; circles: sampling points for mycotoxin quantification; n = 2 samples produced per 40 L initial milk and per type of starter culture; n = 32 samples produced in total.

**Figure SI 2**: Chromatograms obtained for the analysis of TMR spiked with **A):** AFB_1_ [1 µg/kg, retention time 13.9 min] and **B):** CPA [100 µg/kg, retention time 11.5 min] close to the LOQ.

**Figure SI 2**: Chromatograms obtained for the analysis of TMR spiked with **A):** AFB_1_ [1 µg/kg, retention time 13.9 min] and **B):** CPA [100 µg/kg, retention time 11.5 min] close to the LOQ.

**Figure SI 3**: Chromatograms obtained for the analysis of **A)** a milk sample contaminated with 5 ng/L AFM_1_ (spiked) and **B)** a milk sample from the feeding trial with a measured content of 100 ng/L

**Figure SI 4:** HPLC-MS/MS extract ions chromatograms for the qualifier and quantifier transition of AFM_1_ (Q: 329 🡪 273; q: 329 🡪 229; Rt 3.63 min) and three mass transitions expected for the hypothetical AFM_2a_ molecule (MRM1: 347 🡪 329, green; MRM2: 347 🡪 273, grey; MRM3: 347 🡪 229, light blue). **A:** AFM_1_ standard after 4.5 hrs at 60°C in neutral aqueous solution B: AFM_1_ standard after 4.5 hrs at 60°C in 1.5 % citric acid solution.
